# Mendelian randomisation identifies priority groups for prophylactic EBV vaccination

**DOI:** 10.1186/s12879-023-08031-3

**Published:** 2023-02-03

**Authors:** Marisa D. Muckian, James F. Wilson, Graham S. Taylor, Helen R. Stagg, Nicola Pirastu

**Affiliations:** 1 Old Medical School, Usher Institute, University of Edinburgh, Teviot Place, Edinburgh, EH8 9AG UK; 2 MRC Human Genetics Unit, Institute of Genetics and Cancer, Western General Hospital, University of Edinburgh, Crewe Road, Edinburgh, EH4 2XU UK; 3 Institute of Immunology and Immunotherapy, University of Birmingham, Birmingham, B15 2TT UK; 4 Human Technopole, Viale Rita Levi-Montalcini, 1, Area MIND–Cargo 6, 20157 Milan, Italy

**Keywords:** Mendelian randomization, Epstein–Barr virus, Epidemiology, Public health, Vaccination

## Abstract

**Background:**

Epstein Barr virus (EBV) infects ~ 95% of the population worldwide and is known to cause adverse health outcomes such as Hodgkin’s, non-Hodgkin’s lymphomas, and multiple sclerosis. There is substantial interest and investment in developing infection-preventing vaccines for EBV. To effectively deploy such vaccines, it is vital that we understand the risk factors for infection. Why particular individuals do not become infected is currently unknown. The current literature, describes complex, often conflicting webs of intersecting factors—sociodemographic, clinical, genetic, environmental-, rendering causality difficult to decipher. We aimed to use Mendelian randomization (MR) to overcome the issues posed by confounding and reverse causality to determine the causal risk factors for the acquisition of EBV.

**Methods:**

We mapped the complex evidence from the literature prior to this study factors associated with EBV serostatus (as a proxy for infection) into a causal diagram to determine putative risk factors for our study. Using data from the UK Biobank of 8422 individuals genomically deemed to be of white British ancestry between the ages of 40 and 69 at recruitment between the years 2006 and 2010, we performed a genome wide association study (GWAS) of EBV serostatus, followed by a Two Sample MR to determine which putative risk factors were causal.

**Results:**

Our GWAS identified two novel loci associated with EBV serostatus. In MR analyses, we confirmed shorter time in education, an increase in number of sexual partners, and a lower age of smoking commencement, to be causal risk factors for EBV serostatus.

**Conclusions:**

Given the current interest and likelihood of a future EBV vaccine, these factors can inform vaccine development and deployment strategies by completing the puzzle of causality. Knowing these risk factors allows identification of those most likely to acquire EBV, giving insight into what age to vaccinate and who to prioritise when a vaccine is introduced.

**Supplementary Information:**

The online version contains supplementary material available at 10.1186/s12879-023-08031-3.

## Background

Epstein Barr Virus (EBV) is a human herpes virus infecting 95% of the global population. It is associated with multiple cancers including Hodgkin’s and non-Hodgkin’s lymphomas, nasopharyngeal carcinoma and gastric adenocarcinoma, resulting in nearly 164,000 cancer deaths per year. Furthermore, EBV has been associated with the neurological condition multiple sclerosis which affects 2.8 million people globally, particularly in North America, western Europe and Australasia [1, 2]. The burden of disease associated with EBV is such that interest in the development of infection- or disease-preventing vaccines is extensive. In recent years a Phase II trial of an early vaccine candidate to prevent infectious mononucleosis (IM) only reduced symptom severity upon infection [3]. Subsequently, substantial investment has been placed in developing more immunogenic candidates [4]. Efforts have been given a boost by the successful mRNA vaccines developed by Pfizer/BioNTech and Moderna against SARS-CoV-2. Indeed, Moderna currently have a mRNA-based EBV vaccine candidate in clinical development [5].

Our knowledge on the risk factors for the acquisition of EBV is poor, and yet this is critical for two reasons. Firstly, to determine how best to deploy an infection-preventing vaccine. For example, determining the age at which vaccination should take place based on age of exposure to the determined risk factors. Secondly, it is also important that we understand why some individuals remain EBV negative for life and the consequences of this status, given that such vaccines will ‘induce’ a state of potentially lifelong non-infection in millions of people.

Extensive work has been undertaken internationally to determine the risk factors for EBV infection, but these studies have been hindered by the limitations of traditional epidemiology. In a recent systematic review [6], we mapped the highly complex webs of—often contradictory—evidence available to date, and the extent that it is likely to be impacted by unmeasured confounding and reverse causality. Studies to date have largely focused on sociodemographic, dietary, and lifestyle factors, although some of these risk factors are consistently displayed from setting to setting (age being the clearest example). Additionally, a small number of studies have examined genetic susceptibility to EBV infection (the presence or absence of) [7–13]. More recent studies have identified genetic variants associated specifically with levels of antibodies against EBV [9, 10, 14]. One of the issues with improving the evidence is the cost associated with running large, data-rich, cohort studies for which information on all potential risk factors and confounding factors is captured.

Mendelian randomisation (MR) is a technique that takes advantage of genetic data to understand if a putative risk factor of interest is causally associated with a given health outcome [15]. Unlike traditional epidemiological studies, MR eliminates the issues of unmeasured confounding and reverse causality using instrumental variables (IVs), which are genetic variants known to be associated with the risk factor. This means it is possible to accurately determine if a putative risk factor is causally associated with an outcome, provided the assumptions of MR are met and relevant genetic data are available.

This study sought to unravel the Gordian knot of risk factors for the acquisition of EBV, in preparation for the deployment of prophylactic vaccination candidates. We performed a genome-wide associated study (GWAS) to determine the genetic risk factors for EBV infection, followed by an MR to interrogate the published putative non-genetic factors, all within the UK Biobank (UKB; a UK based cohort study of people aged between 40 and 69 years) [16]. Our study demonstrates the power of MR in overcoming the pitfalls of traditional epidemiological approaches, not only for EBV, but also for other complex infectious diseases.

## Methods

In order to perform an MR on the association between the acquisition of EBV infection and different putative risk factors, we undertook the following steps, each of which are laid out in separate sections of the methods. (1) Identify a population of interest for the analysis within which (2) EBV serostatus (as a proxy for infection) had been tested for and (3) which had been genotyped. (4) Identify the putative risk factors of interest for EBV infection from the published literature. (5) Descriptively analyse the population of interest in light of the putative risk factors of interest. (6) Find corresponding existing GWASs to extract instrumental variables (genetic variants known to be associated our putative risk factors of interest. (7) Undertake a GWAS of EBV serostatus and where pre-existing GWASs could not be found for a putative risk factor of interest. (8) Perform MR.

### Study population

UKB is a prospective cohort study of over 500,000 participants recruited in the UK between 2006 and 2010. Participants of the UKB were aged between 40 and 69 years old at the time of recruitment [16].

### Epstein–Barr virus serostatus

A randomly selected subset of 9695 participants in the UKB were subject to serological testing on samples taken at the point of their enrolment into the cohort, including for anti-EBV antibodies [17]. A multiplex serology-based approach, as described by Brenner et al*.* [18], was used for testing. Antibody levels against different EBV antigen targets were expressed as median fluorescence intensity (MFI). (Data were recorded by the UKB as both antibody levels against each antigen and in a binary format (seropositive/seronegative) for overall EBV serostatus if two or more MFI thresholds were met. As EBV is a herpesvirus that establishes a lifelong infection in humans, we used serostatus as a proxy for EBV infection throughout our analyses.

### Genotyping

Participants of the UKB had DNA extracted from samples taken during their initial visit to one of 22 assessment centres. Genotyping was carried out using the Applied Biosystems UK Biobank Axiom Array and the UK BiLEVE array. Autosomal single nucleotide polymorphisms (SNPs) were imputed using a merged reference panel of the Phase 3 1000 Genome Project and UK10K using IMPUTE3. Procedures are described in full by Bycroft et al. [16].

### Exposures to be tested for causal effect on EBV serostatus

**Fig. 1:**
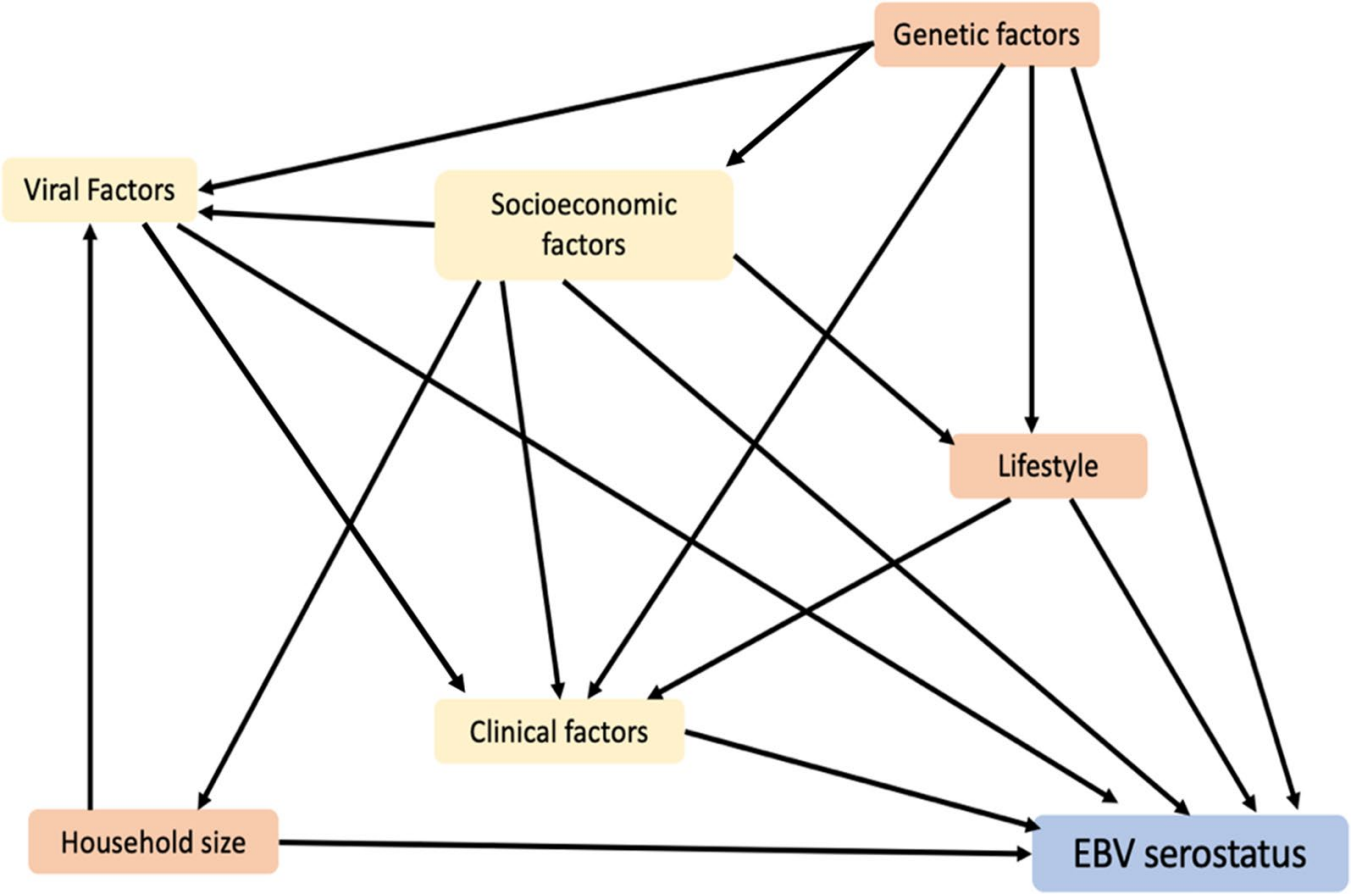
Causal diagram of risk factors for EBV infection. Diagram contains risk factors mapped to broad categories such as household size, lifestyle factors (smoking status), socioeconomic factors (educational attainment), and clinical factors (body mass index, tonsillectomy)

Non-genetic variables to be tested for a putatively causal effect on EBV serostatus through MR were selected based on a review by Winter et al. [6], Six factors (childhood household size, total number of sexual partners, BMI, tonsillectomy, educational attainment, smoking status) were selected on the basis of the balance of evidence within the review being in favour of a putative causal effect (Additional file 1: Table S1) and mapped into a causal diagram (Fig. 1) in broad groups: household size, lifestyle factors (smoking status), socioeconomic factors (educational attainment), genetic factors, clinical factors (BMI, tonsillectomy). The review found coinfection with other several other viruses to be associated with EBV status including: human immunodeficiency virus (HIV), Kaposi’s sarcoma related herpes virus (KSHV), human T-lymphotropic virus (HTLV), CMV, and herpes-simplex 1 (HSV-1). We did not include these in our MR studies due to potential of overlapping genetic variants that may influence both the risk factor infection and EBV.

### Descriptive analysis

A descriptive analysis of the demographics of the overall UKB cohort and the subcohort of individuals with EBV serostatus and who were genomically deemed to be of white British ancestry (see genome-wide association study section of the methods) was carried out using R (v3.6.1) [19]. Qualitative traits are reported as a % total (N). Normality of the quantitative variables was assessed using a Kolmogorov–Smirnov test and non-normally distributed traits were subsequently expressed using the median and interquartile range.

### Exposure instrumental variable selection

Next, IVs for each exposure (genetic variants associated with the exposures i.e., putative risk factors, in this case, SNPs) were selected. From the causal diagram, six putative risk factors were selected. For all risk factors apart from household size, IVs were obtained from previously published GWAS results (Additional file 1: Table S2). For each exposure variable, the largest and most recent GWAS performed in European samples was used. From each exposure GWAS we selected genome wide significant (p < 5 × 10^–8^) and independent SNPs (r^2^ < 0.0001) using the TwoSampleMR package. For total number of siblings GWAS results were not available, thus we performed our own GWAS using the individuals from UKB who did not participate in the serological study (N = 319,209). For total number of siblings, we combined the total number of sisters and total number of brothers variables as reported in the questionnaire of UKB. GWAS methods are described below.

### Genome-wide association study for EBV serostatus

As a preparatory step for the MR, we carried out two GWAS: (1) of EBV serostatus on the subcohort, our MR outcome variable and (2) of household size as measured by total number of siblings, as no IV could be identified from the literature for this putative risk factor. These were carried out on UKB participants with genomic data, including only unrelated individuals and those who were genomically deemed to be of white British ancestry determined using a principal component analysis (PCA) performed by UKB [16]. Genome-wide association was performed using a two-step regression framework, which first created phenotype residuals adjusted for technical genotyping covariates and population structure, before regressing these residuals against SNP dosage. This two-step approximation (GRAMMAR-Gamma) is commonly used in large GWAS of related individuals to reduce the computational burden by fitting a mixed model only once for each phenotype, instead of fitting the model for every SNP [21]. Using a purpose-built GWAS pipeline, two phenotypes were regressed against the fixed effect covariates (sex, age, genotyping batch, array type, and the first 40 principal components (PCs) as calculated by UKB to account for population substructure) [20]. Fixed effect residuals were then further regressed against a random effect covariate of relatedness, based on the variance of the sparse genetic relatedness matrix using FastGWA [21]. Finally, the FastGWA residuals were regressed against genome-wide SNP dosages using RegScan [22]. Genome-wide association was performed using a linear regression model. Genome-wide significant loci were defined using a p-value threshold of 5 × 10^–8^. The resulting SNPs from the total number of siblings GWAS were chosen as IVs for MR analysis and included independent significant SNPs (r^2^ = 0.001, p-value = 5 × 10^–8^).

### Mendelian randomization

**Fig. 2:**
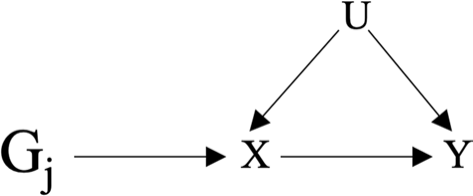
Mendelian randomization. Mendelian randomization uses genetic instruments (G_j_) associated with the exposure (X) of interest as instrumental variables, to determine the causal relationship of X on the outcome (Y) without the influence of confounding. The instruments must not be association with any confounders (U)

MR analysis allows us to determine the causal role that a given exposure (X) has on a given outcome (Y) without the impact of confounding. Genetic instruments- such as SNPs—that directly affect the exposure of interest, can be used as IVs to determine the exposure’s effect on a specific outcome. If individuals with genetic variants associated with the risk factor, have a higher incidence of the outcome, in this case EBV seropositivity, we can conclude that the risk factor is causal for EBV. Genetic variants are valid instruments so long as they are not also associated with the outcome and are not influenced by any confounders (U) (Fig. 2).

For this study we used Two-Sample MR, in which the effects of the SNP on the exposure and the outcome are estimated in two distinct set of samples. The two effect sizes are harmonized, to ensure both the outcome and exposure effects align to the same allele. The effect of the exposure on the outcome is then estimated. This method was used to test if seven (including two measures of smoking status, age at smoking initiation and ever smoking) previously identified putative risk factors had a role in influencing risk of EBV infection. As an outcome, we used our EBV serostatus GWAS. The exposure and outcome data were harmonised before MR was performed. We firstly detected outliers using the RadialMR package and IVW radial function. We then removed these outliers from the harmonised data. To test the validity of our causal inferences we carried out multiple sensitivity analyses. Firstly, using TwoSampleMR, we calculated Cochran’s Q statistic to assess heterogeneity of the genetic instruments. We then checked for directional pleiotropy using the Egger regression function. To ensure no single genetic variant was impacting the causal estimate of our results, we performed a leave one out analysis using TwoSampleMR.

Multivariable MR (MVMR) was planned to assess for correlation of significant factors at the univariable stage using the Mendelian randomization package [23].

## Results

### Descriptive analysis

Of the 9695 individuals within the UKB sub-cohort that underwent serological testing, 8244 (97.3%) had available results for EBV serostatus and were genomically deemed to be of white British ancestry (Additional file 1: Table S3, which also compares the subcohort the overall UKB cohort). Of those 7795 (94.6%) were EBV seropositive. The age and sex distribution within the sub-cohort were similar to that of the main cohort.

### Genome-wide association study

GWAS of EBV serostatus (positive or negative) revealed two independent genome-wide significant loci for EBV serostatus (p ≤ 5 × 10^–8^) (Additional file 1: Fig. S1, Table S4). The first locus was located on chromosome 13 and mapped nearest to *RASA3*, (rs71449058, p = 2.34 × 10^–10^); effect allele C has a protective effect against EBV. The second locus was on chromosome 6 and the nearest gene *PREP* (rs1210063, p = 4.01 × 10^–9^), the effect allele G was found to increase susceptibility to EBV.

### Mendelian randomization

**Fig. 3:**
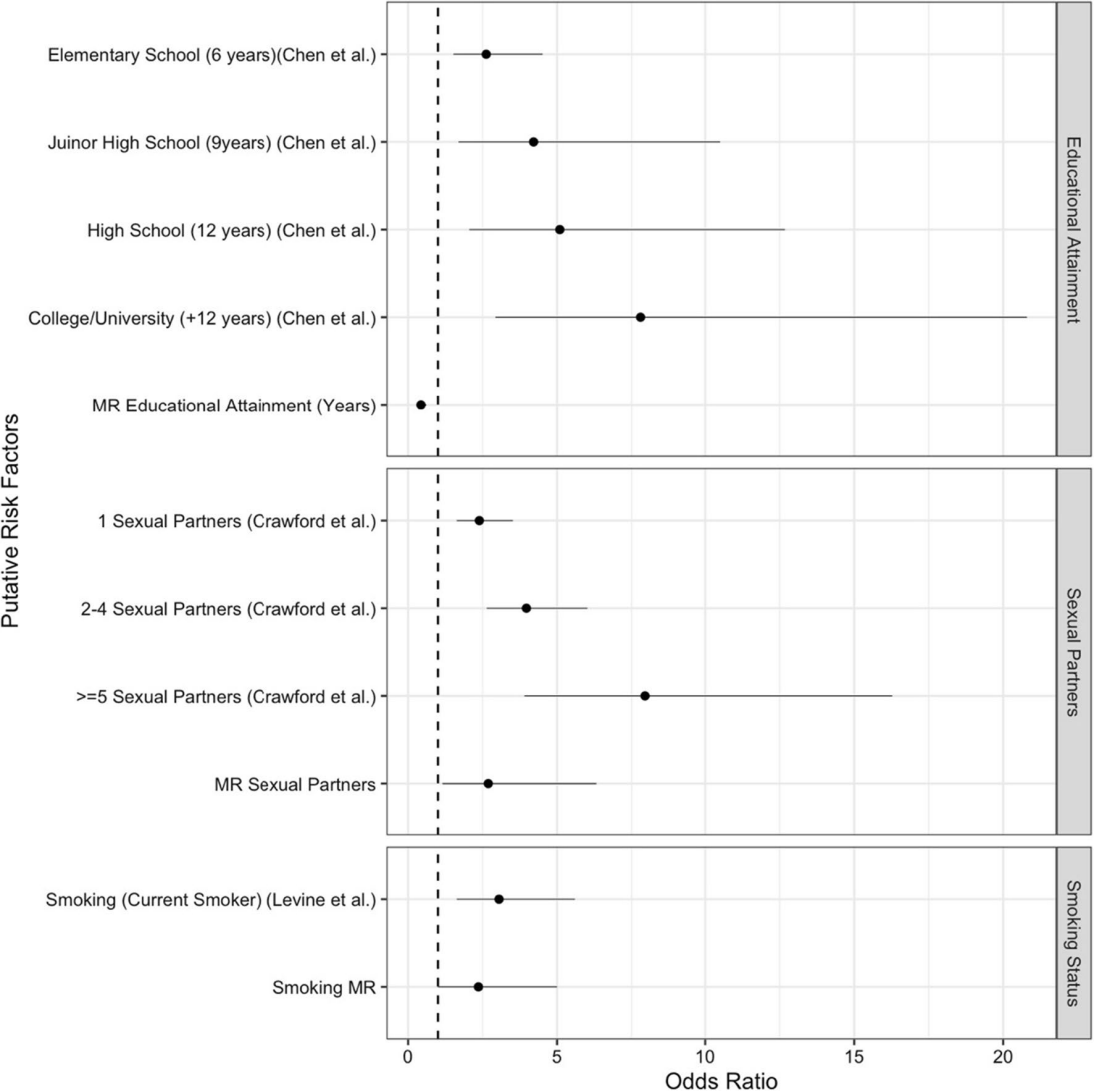
Mendelian randomization results compared to previous observational studies for educational attainment, number of sexual partners and smoking status. Our educational attainment MR compared to an observational study in Taiwan (Baseline = uneducated) by Chen et al. [28]. The sexual partners MR compared to observational effects converted from risk factors from a previous study by Crawford et al. [29] (Baseline = 0). Smoking status MR compared to a study by Levine et al. [30] (Baseline = never smoked). *MR* Mendelian randomization

Taking the results of our GWAS, we next sought to determine if our putative risk factors for EBV serostatus were, in fact, causal. After removal of outlying IVs (Additional file 1: Table S5), univariable MR showed that educational attainment (p = 7.20 × 10^–6^), sexual partners (p = 0.02)*,* and smoking (p = 0.049) were found to be associated with EBV serostatus (Table 1). For each additional year of genetically predicted education (baseline 0 years) the odds of being EBV seropositive decreased (OR = 0.43, 95% CI = 0.30–0.62). Compared to previous studies this we observed the opposite direction of effect (Fig. 3) although effect size was difficult to compare due to differences in exposure measurements. As the total number of sexual partners increased from < 2 partners to between 2 and 5, the odds of being EBV seropositive increased to 2.69 (95% CI = 1.15–6.32), consistent with previous literature. Finally, being a smoker (previous or current) increased the odds of EBV 2.36 times (95% CI = 1.00–5.55). No other putative risk factors were found to be associated with EBV serostatus.

**Table 1:** Univariable Mendelian randomization of putative risk factors for EBV infection

Risk factor	N SNPS	OR (95% CI)	P-value
Total number of siblings	5		0.84
Per increase of 1		1.15 (0.30–4.38)	
Number of sexual partners^a^	61		0.02
< 2		Baseline	
2–5		2.69 (1.15–6.32)	
BMI	464		0.29
Per unit increase		1.14 (0.89–1.46)	
Tonsillectomy	10		0.37
No		Baseline	
Yes		0.01 (5.5 × 10^–7^–220.33)	
Educational attainment (years)	291		7.20 × 10^–6^
0		Baseline	
Per increase of 1		0.43 (0.30–0.62)	
Smoking status	10		0.049
Never		Baseline	
Ever		2.36 (1.00–5.55)	
Age at smoking initiation (years)	1		0.80
Per unit increase		0.92 (0.47–1.79)	

Putative risk factors include, total number of siblings, total number sexual partners (< 2, 2–5, ≥ 5)^a^ (OR only calculated for < 2 and 2–5), BMI, Tonsillectomy (yes/no), Educational attainment (years), smoking status (never/ever), age at smoking initiation (years). Risk factors with p ≤ 0.05 were considered significant

*BMI* body mass index, *CI* confidence intervals, *N* number, *OR* odds ratio, *SNPs* single nucleotide polymorphisms

Sensitivity analyses demonstrated no significant heterogeneity between the estimate from each of the exposure IVs and EBV status while we detected no sign of directional pleiotropy when tested using Egger regression. Finally leave-one-out analysis showed the observed effect was constant and not driven by any single SNP (Additional file 1: Fig. S2a–c).

### Multivariable Mendelian randomization

To determine if education, total number of sexual partners and smoking were independent risk factors for EBV we performed MVMR (Table 2). Results indicated that educational attainment was an independent risk factor for EBV (OR = 0.46, 95% CI = 0.32–0.67, p = 3 × 10^–6^). Smoking also was an independent risk factor (OR = 4.13, 95% CI = 1.51–11.30, p = 0.006). The total number of sexual partners had a similar OR to the univariable analysis [OR = 2.12, 95% CI = (0.66–6.82)], but the result was not statistically significant (p = 0.206). This could be due to fewer IVs in the MVMR being associated with total number of sexual partners, reducing power.

**Table 2:** Multivariable Mendelian randomization of risk factors for EBV infection

Trait	OR (95% CI)	p-value
Educational attainment (years)		3 × 10^–6^
Per increase of 1	0.47 (0.33–0.67)	
Total number of sexual partners		0.21
< 2		
2–5	2.12 (0.66–6.82)	
Smoking status		0.006
Never	Baseline	
Ever	4.13 (1.51–11.30)	

Model adjusted for three risk factors, total number sexual partners (< 2, 2–5, ≥ 5), educational attainment (years), smoking status (yes/no), Risk factors with p ≤ 0.05 were considered significant

*CI* confidence intervals, *OR* odds ratio

## Discussion

In this study, we present the first MR to examine the causality of potential risk factors for the acquisition EBV. Our MR analysis of previously identified risk factors and EBV serostatus demonstrates how MR can be used to unpick the available, at times conflicting, evidence on the complex spectrum of factors that pose a risk for the acquisition of an infectious disease. This is not only the case for EBV, but it also provides a proof of principle for other infectious diseases. In our study we identified two loci (rs1210063 and rs71449058) associated with EBV infection through an initial GWAS and provided evidence that the non-genetic factors educational attainment and number of sexual partners and smoking are likely causally associated with infection.

Examining the loci documented within our GWAS first, previous publications have documented that one of the nearest genes to these loci have previously been discussed in the EBV literature (*RASA3*). This gene locates near viral protein binding sites that may enhance regulation of the EBV lytic cycle [24].

Comparing our findings to previous studies of the genetics of EBV infection, it is interesting to note that such studies have focused primarily on antibody levels. Anti-EBNA-1 levels have been established to associate strongly with the HLA class II region [9–12, 14] and more recently this region was found to be associated with anti-VCA IgG [14]. In a recent publication, Butler-Laporte et al*.* found similar results to our GWAS, despite slight differences in sample selection, the top SNP for EBV seropositivity documented in that publication—rs71437272—showed a similarly strong result in our analysis [13].

While our GWAS results provided insight into the genetic susceptibility component of our causal framework, they also gave us the tools required to untangle the conflicted evidence reported in the literature for EBV risk factors. An increased number of sexual partners and either being or having been a smoker increased risk of EBV. We found that having a higher educational attainment was protective for EBV in univariable MR, in contrast to the results of Chen et al*.* [25], possibly due to differential access to education at the relevant time points in the UK and Taiwan. Given the association in the UK between years spent in education and socioeconomic status, as well as smoking and socioeconomic status, these two findings correlate within our MR. MVMR found smoking and educational attainment to be independent risk factors for EBV status. The direction of the effect of these risk factors is consistent with the previous literature [25–28]. In contrast, BMI, age at smoking initiation, total number of siblings, and having your tonsils removed were not associated with EBV in this MR analysis.

With the recent surge in interest in EBV infection-preventing vaccines, our results present an intriguing insight into the future deployment of such products on the basis of known risk factors for EBV infection. For example, the cost of a future vaccine may limit publicly funded deployment to particularly at-risk groups from EBV associated diseases. There is a known association between EBV acquisition at later life stages, infectious mononucleosis and then cancer [29], as well as a likely strong association between the time point of acquisition and population level socioeconomic status [6]. Thus our documentation of two individual level socioeconomically associated factors (smoking [30] and years in education) as likely causally associated with infection demonstrates an opportunity for targeted deployment of the vaccine to particular population groups. Whilst it is not possible to deploy a vaccine on the basis of a factor such as smoking status, doing so on the basis of enrolment in different levels of education is commonly used for other infectious diseases e.g. meningitis A, C, W, Y.

The core strength of our study is the use of MR to unpicking the previously disagreed upon or at times opposing evidence of causality for the risk factors for an EBV without confounding or reverse causality. Although our study population was restricted to individuals genomically deemed to be of white British ancestry, limiting generalisability. Moreover, only three (3/77) studies used to identify putative EBV risk factors from the review by Winter et al., were from the UK population and two of the observational studies used in comparison to the MR results are from Taiwan (education) and Israel (smoking). EBV seroprevalence and the age by which seroprevalence reaches equilibrium varies between populations [6] and both genetic and non-genetic factors are likely to vary too. Additional studies across populations of different ancestries are required. Data were only available on EBV serostatus at baseline within the UKB, limiting our ability to examine risk factors in temporal proximity to EBV acquisition. UKB, like many population cohorts, is known to not be truly representative of the general population and is particularly enriched for individuals of higher educational status. There is also evidence that EBV infectivity rates differ between EBV strains (type 1 and type 2), and so the viral genome might influence conversion rates [31]. However, no viral genetic data were available in our study. The previous observational studies used to test our hypothesis also did not distinguish between EBV type. Finally, our study had limited power due to 95% of individuals being EBV seropositive.

Despite these limitations, we found MR to be a powerful tool to clearly define a core set of risk factors for the acquisition of EBV. These factors are informative for future vaccine deployment and should be measured and then adjusted for in analyses of the acquisition of EBV. Other infectious diseases for which MR would be similarly useful include respiratory syncytial virus (RSV). A review of the putative risk factors for RSV and acute lower respiratory infections from 2015 described the huge variation between studies in how risk factors are measured, and which confounders are adjusted for [32]. The effect estimates in these studies were thought to be impacted substantially by confounding and the biased measurement of putative risk factors; MR has the potential to solve this issue by pinpointing which risk factors to measure and adjust for.

The effective deployment of anti-EBV vaccines will not be possible without a better grasp on the acquisition of infection than has been provided by epidemiological studies to date. Using MR, we demonstrate a low-cost and effective way of untangling the published literature and pinpoint critical factors to consider when vaccine candidates come onto the market.

## Conclusions

Given the likelihood that an EBV vaccine will become available, we have identified key sociodemographic risk factors that will aid identification of target groups for priority vaccination. We also ruled out several disputed factors identified in previous literature, implicating the usefulness of MR in the study of infectious disease risk factors.

## Supplementary Information

**Additional file 1: Table S1.** Putative non-genetic risk factors for Epstein Barr virus infection to be explored in the Mendelian randomisation. **Table S2.** Table of GWAS studies used in Mendelian randomization analysis. **Table S3.** Demographic and clinical baseline data for the UK Biobank cohort versus the sub-cohort for analysis. **Figure S1.** Manhattan plot of EBV serostatus loci. **Table S4.** Significant GWAS hits of EBV serostatus. **Table S5.** Table of heterogeneity statistics from TwoSampleMR package and number of outliers detected. **Figure S2.** (a) Leave one out analysis for educational attainment. (b) Leave one out analysis for lifetime number of sexual partners. (c) Leave one out analysis for age at smoking initiation.

## Data Availability

The source data are available to researchers upon application to UK Biobank using the UKBiobank data access process (http://www.ukbiobank.ac.uk/register-apply/). Full GWAS summary statistics for our EBV serostatus and total number of siblings can be found at 10.7488/ds/3797. Summary statistics for smoking status and age of smoking initiation can be downloaded from (https://conservancy.umn.edu/handle/11299/201564). Summary statistics for lifetime number of sexual partners (ukb-b-4256), tonsillectomy (ukb-b-15096), BMI (ieu-b-40) and educational attainment (ieu-a-1239) can be downloaded from the open GWAS project (https://gwas.mrcieu.ac.uk/).
