# Supplementary material for: Mendelian randomisation identifies priority groups for prophylactic EBV vaccination
Source: BMC Infect Dis. 2023 Feb 3;23:65. doi: 10.1186/s12879-023-08031-3 (PMC9896437; doi:10.1186/s12879-023-08031-3)
Supplement: Supplementary file 1 — Additional file 1: Table S1. Putative non-genetic risk factors for Epstein Barr virus infection to be explored in the Mendelian randomisation. Table S2. Table of GWAS studies used in Mendelian randomization analysis. Table S3. Demographic and clinical baseline data for the UK Biobank cohort versus the sub-cohort for analysis. Figure S1. Manhattan plot of EBV serostatus loci. Table S4. Significant GWAS hits of EBV serostatus. Table S5. Table of heterogeneity statistics from TwoSampleMR package and number of outliers detected. Figure S2. (a) Leave one out analysis for educational attainment. (b) Leave one out analysis for lifetime number of sexual partners. (c) Leave one out analysis for age at smoking initiation. [file 12879_2023_8031_MOESM1_ESM.docx]

***Mendelian randomisation identifies priority groups for prophylactic EBV vaccination.***

**Additional Figures:**

[**Figure S1 Manhattan plot of EBV serostatus loci**. Manhattan plot showing the strength of the associated -log10 (pvalue) against the chromosome location of the SNPs. Genome wide significance is represented by the red line (5x10-^8^) 5](#_Toc94601957)

[**Figure S2a) Leave one out analysis for educational attainment** Educational attainment leave one out sensitivity analysis removed each instrumental variable from the Mendelian randomization, no single instrument was driving the effect estimate. 8](file:////Users/mmuckian/Documents/Manuscripts/Supplementary_01_31_2022.docx#_Toc94601958)

[**Figure S2b) Leave one out analysis for lifetime number of sexual partners** sexual partners leave one out sensitivity analysis removed each instrumental variable from the Mendelian randomization, no single instrument was driving the effect estimate. 9](#_Toc94601959)

[**Figure S2c) Leave one out analysis for age at smoking initiation** Smoking initiation leave one out sensitivity analysis removed each instrumental variable from the Mendelian randomization, no single instrument was driving the effect estimate. 10](#_Toc94601960)

**Additional Tables:**

[**Table S3 Demographic and clinical baseline data for the UK Biobank sub-cohort.** Abbreviations, BMI – body mass index, EBV – Epstein-Barr Virus, IQR – interquartile range 4](#_Toc94601914)

[**Table S4 – Significant GWAS hits of EBV serostatus.** Table showing the four loci that were significant after our genome wide association study (GWAS) of EBV serostatus and their nearest mapped gene. 6](#_Toc94601915)

[**Table S5 Table of heterogeneity statistics from TwoSampleMR package and number of outliers detected**. TwoSampleMR was used to test for heterogeneity for each risk factor, Outliers from each analysis were also detected and removed. 7](#_Toc94601916)

**Table S1 Putative non-genetic risk factors for Epstein Barr virus infection to be explored in the Mendelian randomisation.** Six factors were identified to have a sufficient balance of evidence from Winter et al.^1^ to be considered as putative risk factors in our Mendelian randomisation analysis: total number in household, total number of sexual partners, BMI, tonsillectomy, educational attainment, and smoking status. Abbreviations: BMI – body mass index, CI – confidence intervals, EBV – Epstein Barr Virus, OR – odds ratio, Ref – references, UK- United Kingdom, USA – United States of America

| Risk Factor | Author | Year | Country | Summary | Ref |
| --- | --- | --- | --- | --- | --- |
| Total number of siblings | Levine *et al.*  Jansen *et al.* | 2012  2016 | Israel  The Netherlands | Increase in seroprevalence with increased number of siblings  EBV seropositive children had 2 or more siblings (OR= 1.35; 95% CI 1.05-1.74) | ^2,3^ |
| Total number of sexual partners | Crawford *et al.* | 2002 | UK | Prevalence of seropositivity is increased amongst those who are sexually active | ^4^ |
| BMI | Thjodleifsson *et al.*  Bertrand *et al.*  Dowd *et al.*  Spielman *et al.* | 2008  2010  2013  2014 | Iceland, Sweden, Estonia  USA  USA  USA | Lower seroprevalence with increased BMI | ^5–8^ |
| Tonsillectomy | Durovic *et al.* | 2013 |  | Lower seroprevalence amongst those with history of tonsillectomy | ^9^ |
| Educational attainment | Chen *et al.* | 2015 | Taiwan | Higher educational level associated with higher seropositivity rate | ^10^ |
| Smoking status | Levine *et al.*  Xu *et al.* | 2012  2012 | Israel  China | Seropositivity associated with smokers compared to non-smokers (OR, 3.05; 95% CI, 1.63-5.60)  Smoking associated with EBV seropositivity | ^2,6,11^ |

**Table S2 Table of GWAS studies used in Mendelian randomization analysis.** Studies chosen for Mendelian randomization (MR) analyses. BMI, tonsillectomy, number of sexual partners, educational attainment summary statistics and instruments were extracted from the available outcomes within the TwoSampleMR package. Cigarettes per day and age at smoking initiation summary statistics were downloaded as per the publication’s instructions. Number of siblings instruments were extracted from GWAS results performed in house. BMI – body mass index, GWAS- genome wide association study.

^a^ summary statistics not from published article

| Trait | Author | Year | Sample Size |
| --- | --- | --- | --- |
| BMI | Yengo *et al.*^12^ | 2018 | 681,275 |
| Tonsillectomy | Elsworth *et al.^a^* | 2018 | 462,933 |
| Number of Sexual Partners | Elsworth *et al.^a^* | 2018 | 378,882 |
| Educational Attainment | Lee *et al.*^13^ | 2018 | 766,345 |
| Age at smoking initiation | Liu *et. al*^14^ | 2019 | 1,232,091 |
| Smoking initiation | Liu *et al.*^14^ | 2019 | 337,334 |
| Number of Siblings | In house GWAS | - | 487,409 |

| Variable | UKBiobank Cohort | | EBV serology results present and genomically deemed to be of white British ancestry (subcohort) | | |
| --- | --- | --- | --- | --- | --- |
|  | N | Column % | N | Column % |  |
| TOTAL | 502,616 | 100.0 | 8,244 | 100.0 |  |
| Age (years) | Median (IQR): 58.0 (51.0-64.0) |  | Median (IQR): 57.3 (51.0-64.0) |  |  |
|  | 502,614 | 99.9 | 8,244 | 100 |  |
| Missing data | 2 | 0.0004 | 0 | 0 |  |
|  |  |  |  |  |  |
| Sex |  |  |  |  |  |
| Male | 229,163 | 45.6 | 3,618 | 43.9 |  |
| Female | 273,453 | 54.4 | 4,626 | 56.1 |  |
| Total Number of Siblings | Median (IQR): 2 (1-3)  493,228 | 98.1 | Median (IQR): 2 (1-3)  8,235 | 99.9 |  |
| Missing data | 9,388 | 1.9 | 9 | 0.1 |  |
| Total Number of Sexual Partners | Median (IQR): 3 (1-6) |  | Median (IQR): 3 (1-6) |  |  |
| <2 | 116,685 | 23.2 | 2,019 | 24.5 |  |
| 2-5 | 130,739 | 26.0 | 2,186 | 26.5 |  |
| ≥5 | 157,745 | 31.4 | 2,616 | 31.7 |  |
| Missing data | 97,447 | 19.4 | 1,423 | 17.3 |  |
| BMI | Median (IQR): 26.7 (24.1-29.9)  499,511 | 99.4 | Median (IQR): 27.28 (24.0-29.7)  8,219 | 99.7 |  |
| Missing data | 3,105 | 0.6 | 25 | 0.3 |  |
| Tonsillectomy |  |  |  |  |  |
| Yes | 1,000 | 0.2 | 13 | 0.2 |  |
| No | 501,616 | 99.8 | 8,231 | 99.8 |  |
| Educational Attainment (years) | Median (IQR): 15(13-20)  492,473 | 98.0 | Median (IQR): 15 (13-20)  8,173 | 99.1 |  |
| Missing data | 10,143 | 2.0 | 71 | 0.9 |  |
| Smoking Status |  |  |  |  |  |
| Current | 52,885 | 10.5^a^ | 804 | 9.8 |  |
| Previous | 173,091 | 34.4 | 2,894 | 35.1 |  |
| Never | 273,588 | 54.4 | 4,511 | 54.7 |  |
| Missing data | 3,052 | 0.6 | 35 | 0.4 |  |

**Table S3 Demographic and clinical baseline data for the UK Biobank cohort versus the sub-cohort for analysis.**  Abbreviations, BMI – body mass index, EBV – Epstein-Barr Virus, IQR – interquartile range

^a^Does not add to 100% due to rounding

**Figure S1 Manhattan plot of EBV serostatus loci**. Manhattan plot showing the strength of the associated -log10 (p value) against the chromosome location of the SNPs. Genome wide significance is represented by the red line (5x10-^8^)
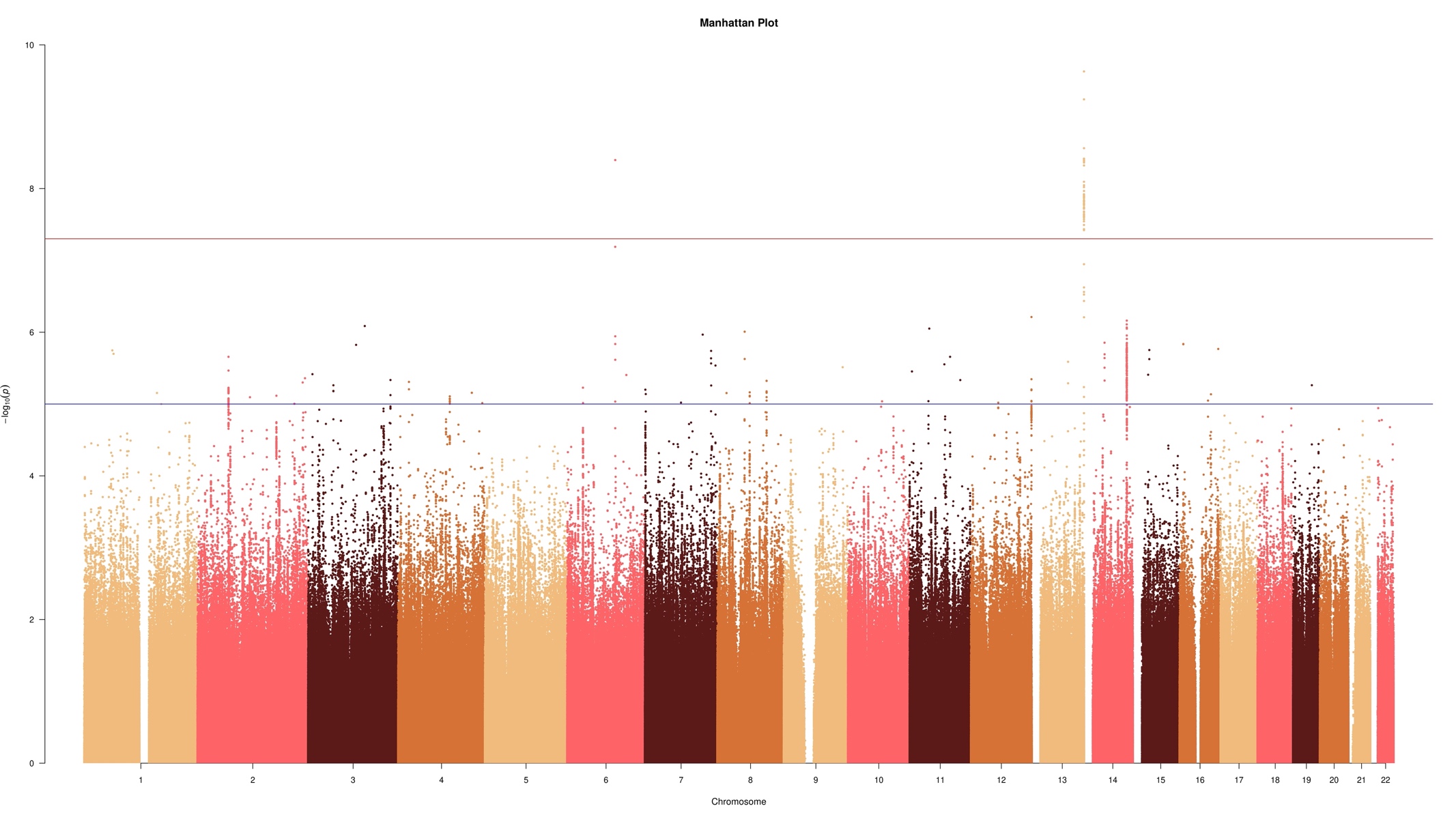


**Table S4 – Significant GWAS hits of EBV serostatus.** Table showing the two loci that were significant after our genome wide association study (GWAS) of EBV serostatus and their nearest mapped gene. Freq- frequency.

| SNP | Chr | Start | End | Pos | A1 | A0 | Beta | SE | P Value | Freq1 | R^2^ | Nearest Gene |
| --- | --- | --- | --- | --- | --- | --- | --- | --- | --- | --- | --- | --- |
| rs1210063 | 6 | 105712424 | 105784121 | 105770008 | G | A | 0.04 | 0.007 | 4.01x10^-09^ | 0.941 | 0.004 | PREP |
| rs71449058 | 13 | 114639490 | 114882724 | 114838361 | C | T | -0.10 | 0.016 | 2.34x10^-10^ | 0.0148 | 0.005 | RASA3 |

**Table S5 Table of heterogeneity statistics from TwoSampleMR package and number of outliers detected**. TwoSampleMR was used to test for heterogeneity for each risk factor, Outliers from each analysis were also detected and removed.

| Trait | Cochran’s Q Statistic | P Value | Outliers removed |
| --- | --- | --- | --- |
| Body mass index | 365.0263 | 0.9996492 | 28 |
| Tonsillectomy | 3.932883 | 0.7874748 | 1 |
| Total number of sexual partners | 44.02451 | 0.9124796 | 1 |
| Educational attainment | 238.3304 | 0.9866870 | 16 |
| Age at smoking initiation | 12.04674 | 0.1491306 | 0 |
| Cigarettes per day | 6.039483 | 0.5351467 | 0 |
| Number of siblings | 3.264745 | 0.3525798 | 0 |


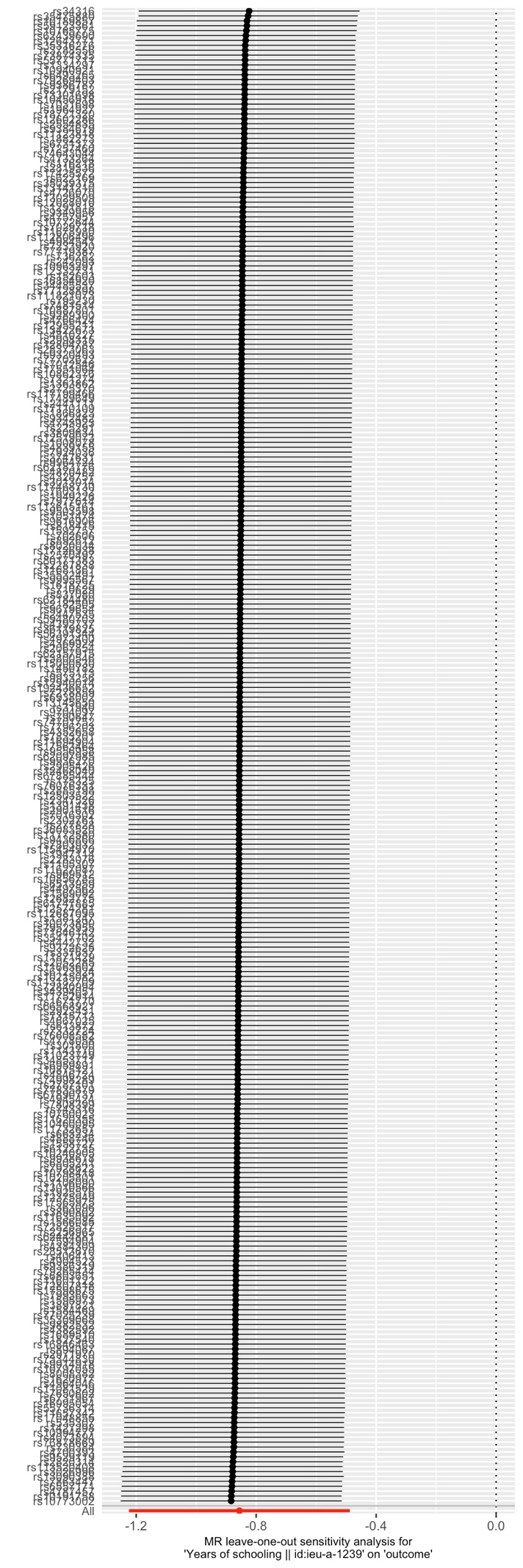


**Figure S2a) Leave one out analysis for educational attainment.** Educational attainment leave one out sensitivity analysis removed each instrumental variable from the Mendelian randomization, no single instrument was driving the effect estimate.

**Figure S2b) Leave one out analysis for lifetime number of sexual partners.** Sexual partners leave one out sensitivity analysis removed each instrumental variable from the Mendelian randomization, no single instrument was driving the effect estimate.


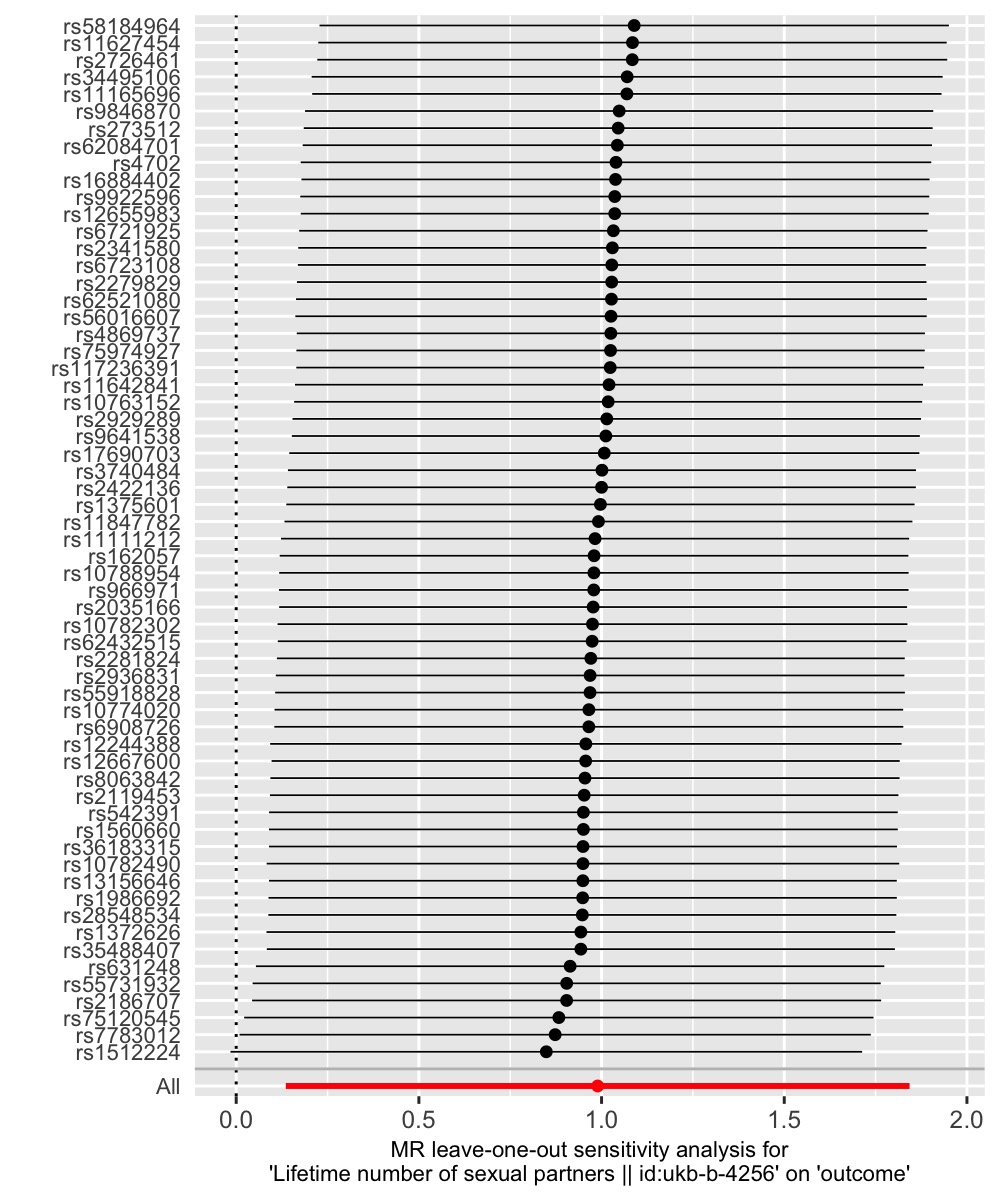


**Figure S2c) Leave one out analysis for age at smoking initiation.** Smoking initiation leave one out sensitivity analysis removed each instrumental variable from the Mendelian randomization, no single instrument was driving the effect estimate.


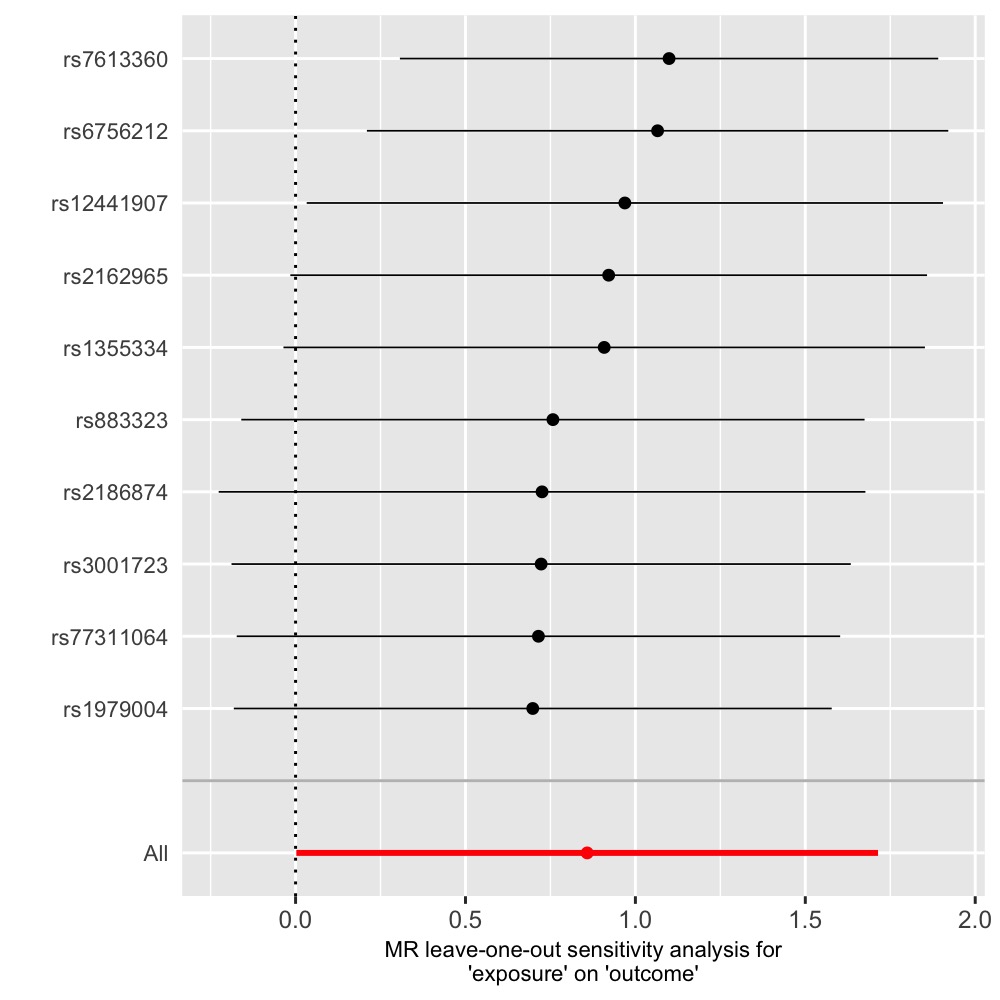


**References**

1 Winter JR, Jackson C, Lewis JE, Taylor GS, Thomas OG, Stagg HR. Predictors of Epstein-Barr virus serostatus and implications for vaccine policy: A systematic review of the literature. *J Glob Health*; **10**. DOI:10.7189/jogh.10.010404.

2 Levine H, Balicer RD, Rozhavski V, *et al.* Seroepidemiology of Epstein−Barr virus and cytomegalovirus among Israeli male young adults. *Annals of Epidemiology* 2012; **22**: 783–8.

3 Jansen MAE, Heuvel D van den, Bouthoorn SH, *et al.* Determinants of Ethnic Differences in Cytomegalovirus, Epstein-Barr Virus, and Herpes Simplex Virus Type 1 Seroprevalence in Childhood. *The Journal of Pediatrics* 2016; **170**: 126-134.e6.

4 Crawford DH, Swerdlow AJ, Higgins C, *et al.* Sexual History and Epstein-Barr Virus Infection. *J Infect Dis* 2002; **186**: 731–6.

5 Bertrand KA, Birmann BM, Chang ET, *et al.* A prospective study of Epstein-Barr virus antibodies and risk of non-Hodgkin lymphoma. *Blood* 2010; **116**: 3547–53.

6 Dowd JB, Palermo T, Brite J, McDade TW, Aiello A. Seroprevalence of Epstein-Barr Virus Infection in U.S. Children Ages 6-19, 2003-2010. *PLoS One* 2013; **8**. DOI:10.1371/journal.pone.0064921.

7 Spielmann G, Johnston CA, O’Connor DP, Foreyt JP, Simpson RJ. Excess body mass is associated with T cell differentiation indicative of immune ageing in children. *Clin Exp Immunol* 2014; **176**: 246–54.

8 Thjodleifsson B, Olafsson I, Gislason D, Gislason T, Jögi R, Janson C. Infections and obesity: A multinational epidemiological study. *Scandinavian Journal of Infectious Diseases* 2008; **40**: 381–6.

9 Durovic B, Gasser O, Gubser P, *et al.* Epstein-Barr Virus Negativity among Individuals Older than 60 Years Is Associated with HLA-C and HLA-Bw4 Variants and Tonsillectomy. *J Virol* 2013; **87**: 6526–9.

10 Chen C-Y, Huang K-YA, Shen J-H, Tsao K-C, Huang Y-C. A Large-Scale Seroprevalence of Epstein-Barr Virus in Taiwan. *PLoS One* 2015; **10**. DOI:10.1371/journal.pone.0115836.

11 Xu F-H, Xiong D, Xu Y-F, *et al.* An epidemiological and molecular study of the relationship between smoking, risk of nasopharyngeal carcinoma, and Epstein-Barr virus activation. *J Natl Cancer Inst* 2012; **104**: 1396–410.

12 Yengo L, Sidorenko J, Kemper KE, *et al.* Meta-analysis of genome-wide association studies for height and body mass index in ∼700000 individuals of European ancestry. *Hum Mol Genet* 2018; **27**: 3641–9.

13 Lee JJ, Wedow R, Okbay A, *et al.* Gene discovery and polygenic prediction from a genome-wide association study of educational attainment in 1.1 million individuals. *Nat Genet* 2018; **50**: 1112–21.

14 Liu M, Jiang Y, Wedow R, *et al.* Association studies of up to 1.2 million individuals yield new insights into the genetic etiology of tobacco and alcohol use. *Nat Genet* 2019; **51**: 237–44.
